# Retraction: Fatal Prion Disease in a Mouse Model of Genetic E200K Creutzfeldt-Jakob Disease

**DOI:** 10.1371/journal.ppat.1012774

**Published:** 2024-12-11

**Authors:** The PLOS Pathogens Editors

After the Correction was published for this article [1, 2], additional concerns were raised. In following up on the additional issues PLOS also re-examined the results and data reported in the published article and correction. The following issues were noted:

- The wt lane of the originally published Fig 2E [1] appears to be discontinuous with the other lanes. Although the data provided with the Correction notice previously issued on this article [2] appear to support the results, they do not resolve the concerns pertaining to the integrity of the originally published panel. In addition, editorial review of the underlying data provided for Fig 2E [2] suggests that the raw blot provided for this figure appears to be a composite of two images.
- The Correction notice previously issued on this article [2] discloses that panels presented in Fig 5 were prepared using spliced blots and provided original data that appear to support the results presented in panels A-C. However, the explanation and data provided in [2] did not directly address or resolve concerns about the integrity of the Fig 5C total+PK αPrP mAb 6H4 blot in [1]. In addition there appear to be vertical splice lines in Fig 5D [1] which were neither addressed or resolved in [2].
- There appear to be irregularities suggestive of splice lines in the background of Fig 7C, and the lanes in the published Fig 7C panel do not appear to match the underlying data provided in the Correction notice previously issued on this article [2]. Also, the image data provided in [2] suggest that the results reported in lanes 1–2 and lanes 3–8 originated from different blots.
- There appear to be similarities within and between the following panels:
  ○ The Fig 6 Anti Prp pAb RTC WT panel and Anti PrP pAb RVC WT panel appear similar. In addition, for both panels lanes 5–8 appear similar to lanes 9–10 with aspect ratio altered.
  ○ Lanes 3–4 and lanes 9–10 of the Fig 6 Anti Prp pAb RTC TgMHu_2_ME200K/ko panel appear similar
  ○ The Fig 6 Anti Prp pAb RTC Mouse RML panel of this article [1] and the Fig 4 mAB 6H4 Sc(RML) Mo panel of [3, corrected in 4, retracted in 5] appear similar.

The issues were not resolved in follow-up discussions.

Regarding the overlap between the results in Fig 6 of this article and Fig 4 of [3, corrected in 4, retracted in 5], the corresponding author states that these panels are the same because they represent the same results. Vertical irregularities suggestive of splice lines were observed in both panels, and the corresponding author provided the data underlying the published results. Upon editorial review of the underlying data, PLOS noted that the underlying data provided for the Anti Prp pAb RTC Mouse RML panel confirm this panel was inappropriately manipulated.

In light of the above concerns that raise concerns about the reliability and integrity of the published results, the *PLOS Pathogens* Editors retract this article.

RG did not agree with the retraction. YFL, ZM, TC, KF, GGK, HB, and DA either did not respond directly or could not be reached.
